# Breast lesions with atypia in percutaneous biopsies, managed with surgery in the last 10 years

**DOI:** 10.3332/ecancer.2019.923

**Published:** 2019-04-11

**Authors:** Mitzy Carrillo, Gregorio Maturana, Cristóbal Maiz, Diego Romero, Francisco Domínguez, David Oddó, Alejandra Villarroel, Dravna Razmilic, María Elena Navarro, Augusto León, César Sánchez, Mauricio Camus

**Affiliations:** 1 Department of Surgical Oncology, Pontificia Universidad Católica de Chile, Santiago, 8330024, Chile; 2 School of Medicine, Pontificia Universidad Católica de Chile, Santiago, 8330024, Chile; 3 Oncological and Head and Neck Surgery, Hospital Sótero del Río, Santiago, 8207257, Chile; 4 Department of Anatomical Pathology, Pontificia Universidad Católica de Chile, Santiago, 8330024, Chile; 5 Department of Breast Radiology, Pontificia Universidad Católica de Chile, Santiago, 8330024, Chile; 6 Department of Hematology-Oncology, Pontificia Universidad Católica de Chile, Santiago, 8330024, Chile

**Keywords:** breast lesions with atypia, flat epithelial atypia, atypical ductal hyperplasia, atypical lobular hyperplasia, lobular carcinoma in situ

## Abstract

**Introduction:**

The optimal management of breast lesions with atypia (BLA), detected in percutaneous biopsies after screening mammograms, is a controversial issue. The aim of this paper is to compare histological diagnosis by percutaneous biopsy with the results of the surgical biopsy of these lesions and to analyse the changes to clinical approach this would imply.

**Method:**

A retrospective study was carried out on patients operated on between June 2007 and June 2017 with a diagnosis of BLA. One hundred and forty-seven patients were identified with a pre-operative diagnosis of flat epithelial atypia (FEA), atypical ductal hyperplasia (ADH), atypical lobular hyperplasia, lobular carcinoma *in situ* and other atypia.

**Results:**

The average age at diagnosis of BLAs was 52 ± 9.4 years. Radiologically, the lesions presented as microcalcifications in 79%, nodules in 15.6% and other lesions 5.4%. 73.5% of these were biopsied by means of digital stereotaxis. All of the patients analysed underwent a partial mastectomy. Changes in a biologically high-risk lesion were observed in 26.5% of the surgical specimens, of which 75.5% corresponded with ADH and FEA. In the percutaneous biopsies consistent with ADH (40.1%), ductal carcinoma was discovered in 6.8% (5.1% *in situ* and 1.7% invasive), which implied specific, multi-disciplinary management. Of the FEAs, 84.8% required a second treatment (surgery and/or hormone therapy ± radiotherapy, depending on whether it concerned FEA 59.6%, ADH 21.2% or ductal carcinoma *in situ* 3.8%).

**Conclusion:**

These data show the clinical relevance in the diagnosis of ADH and FEA in percutaneous biopsies. For the diagnosis of FEA in particular, the associated risk of biologically high-risk lesions and ductal carcinoma is made evident.

## Introduction

Breast cancer is the most common neoplasia in women globally, regardless of race or ethnicity, representing 16% of the cancers that affect women [1]. On account of this, healthcare centres, nearly the world over, have found it necessary to implement screening programmes for breast cancer.

**Figure 1.**
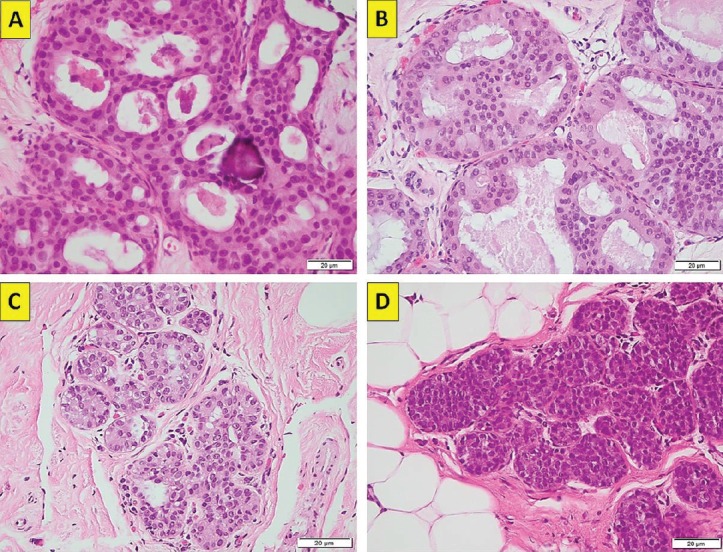
(a): ADH. Cribiform growth pattern with calcifications (haematoxylin-eosin ×40, original magnification). (b): ADH. Cribiform growth pattern with overlapping nuclei (haematoxylin-eosin ×40, original magnification). (c): ALH. Lobule with less than 50% of the glandular epithelium replaced by atypical cell (haematoxylin-eosin ×40, original magnification). (d): in situ. Lobule with classical type and mild glandular distension (haematoxylin-eosin ×40, original magnification). (Original photographs courtesy of Dr David Oddó.)

The rise in investigation by means of mammogram screening has generated an increase of between two and four times the diagnosis of benign breast disease (BBD) and 1.6 times the diagnosis of invasive ductal mammary carcinoma [2]. This would indicate that not all BBD will progress to become malignant lesions. This fact seems to be corroborated by a study that determined, after 12 years of follow-up, that up to 20% of the patients diagnosed with atypical ductal hyperplasia (ADH, Figure 1a and b) or atypical lobular hyperplasia (ALH, Figure 1c) eventually progressed to invasive breast cancer, regardless of the therapeutic or preventive management they had received [3].

**Figure 2.**
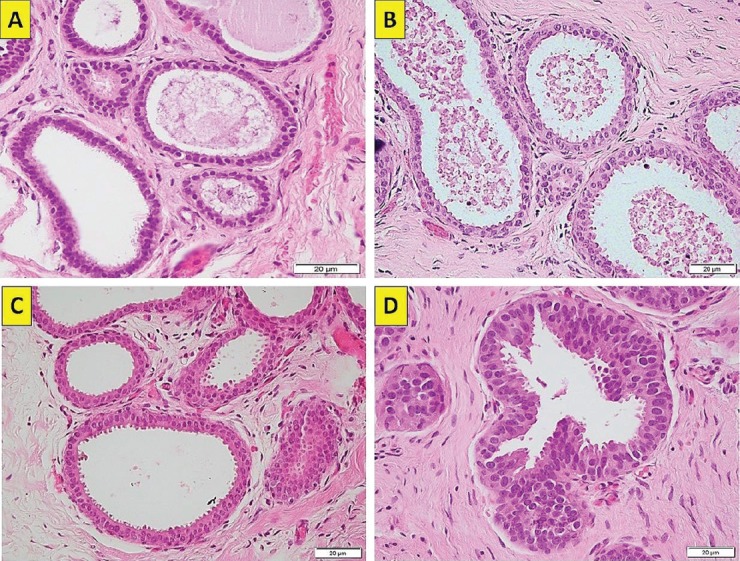
(a): FEA (atypical columnar cell change). Enlarged terminal duct–lobular with monomorphic atypical cells (haematoxylin-eosin ×40, original magnification). (b): FEA (atypical columnar cell change). Enlarged terminal duct–lobular with atypical cells and variant nuclear morphology (haematoxylin-eosin ×40, original magnification). (c): FEA (atypical columnar cell change). Enlarged terminal duct–lobular with monomorphic atypical cells (haematoxylin-eosin ×40, original magnification). (d): FEA (atypical columnar cell change). Enlarged terminal duct–lobular with monomorphic atypical cells (haematoxylin-eosin ×40, original magnification). (Original photographs courtesy of Dr David Oddó.)

The term BBD refers to a rather heterogeneous group of non-malignant breast lesions that are classified into non-proliferative, proliferative and proliferative with atypia. Breast lesions with atypia (BLA) include ADH, ALH and flat epithelial atypia (FEA, Figure 2a–d) or columnar changes with atypia, that all in all comprise close to 10% of all percutaneous biopsies [1].

The optimal management of BLAs detected in percutaneous biopsies after a screening mammogram is a controversial issue, which at present does not appear to have one sole answer with regard to subsequent management. This can consist of strict monitoring, amplification of the surgical resection margin to avoid a misdiagnosis of cancer or in ordering chemoprophylaxis with hormone therapy.

The heterogeneity that BLAs present is demonstrated, for example, in the relative risk (RR), each of these poses of presenting a subsequent breast cancer. Thus, ADH clearly represents a high-risk lesion, with an RR of 4–5, while FEA presents a rather uncertain significance with regard to its risk to present breast cancer [1].

It is important to establish indicators of common consensus to be able, in the future, to define the appropriate management of BLA. In particular, if we consider that the age group most affected by benign mammary pathology is young- or middle-aged people, an incorrect diagnosis or erroneous management of at-risk conditions can significantly impact a patient’s prognosis for survival. In the case of the latter, if BLA are indeed potential markers of future carcinoma, they could also be indicators of concomitant carcinoma that could go undetected due to the small size of the sample taken in the biopsy.

When a clinician finds himself or herself confronted with a biopsy in which a BLA is identified, the decision to recommend surgical excision depends on various factors, among them being the radiological-pathological concordance and the risk of progression that can be attributed to said lesion.

The aim of this paper is to compare histological diagnosis by percutaneous biopsy with the results of a surgical biopsy of these lesions in a cohort of patients having undergone said procedures in the Clinical Hospital of the Pontifical Catholic University of Chile (Pontificia Universidad Católica de Chile). The change in clinical behaviour that the definitive biopsy implicates was also analysed. In this way, we are trying to contribute information on the concordance between BLAs encountered in percutaneous biopsy and the results of the surgical specimen, which in future could assist in giving clinical guidance, in regard to the management of this type of lesion, avoiding their over-treatment or insufficient treatment.

## Materials and methods

A retrospective cohort study was conducted which included all the women who underwent percutaneous biopsy of radiologically suspicious lesions (Breast Imaging Reporting and Data System (BI-RADS) 4 or 5), between 2007 and 2017 in our centre. The assessment and breast imaging reports (mammograms, breast ultrasounds, tomosynthesis, mammograms with a contrast agent and magnetic resonance imaging of the breast) were conducted by the same group of radiologists who were experts in breast pathology.

The patients selected were those with a finding of BLA in the pathology reports from their percutaneous biopsies. Considered as BLA were; the histological diagnoses corresponding to FEA, ADH, ALH, lobular carcinoma *in situ*, (LCIS, Figure 1d) and other less frequent diagnoses, such as papillary lesions with atypia, complex sclerosing lesion with atypia and apocrine metaplasia with atypia. The entirety of the biopsies was performed, analysed and reported by a team of pathologists specialising in breast pathology.

Of all those patients with BLA in their percutaneous biopsy, we selected those that subsequently went through a partial mastectomy and therefore had a surgical biopsy of the lesion. Epidemiological background and monitoring of these patients within the UC Christus Health Network (la Red de Salud UC Christus) were obtained from the electronic medical history.

Descriptive statistics analyses were performed using averages and percentages. The data were tabulated and analysed using the Microsoft Excel programme.

## Results

A total of 147 patients were analysed, on whom percutaneous biopsy was performed between June 2007 and June 2017 and who also subsequently went through surgical resection.

**Figure 3.**
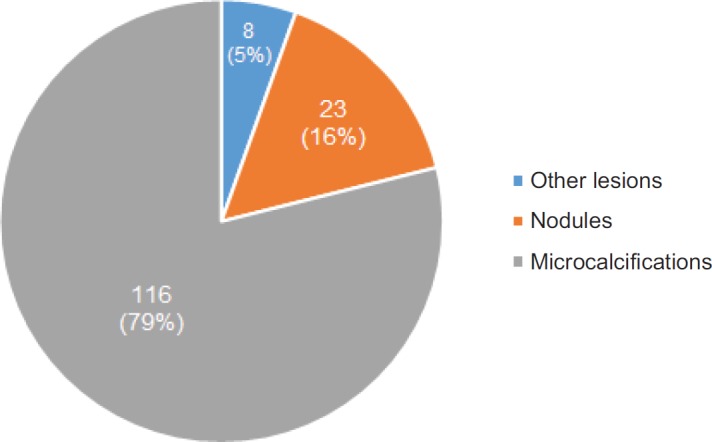
Most frequent imaging findings.

The average age at the time of the diagnosis of BLA was 52.0 ± 9.4 years of age. Radiologically, the lesions presented as; microcalcifications in 79% of cases, nodules in 16% and in the remaining 5% as other lesions, among which are described radial scar and architectural distortion (Figure 3). In terms of laterality, 53% of the lesions were found in the left breast, 46% in the right breast and in only one case, in relation to apocrine metaplasia with atypia, the finding was bilateral.

The method for obtaining the percutaneous biopsy in 73.5% of cases was by digital stereotaxis with a 14 G Suros needle and in the remaining 26.5% by 16 G core needle biopsy using ultrasound. This is due to the high prevalence of microcalcifications as a finding of BLA.

Of all the BLAs diagnosed by percutaneous biopsy, the most prevalent was ADH in 40.1% of cases, followed by FEA in 35.4% and in the smallest proportion ALH, LCIS and other atypia. In subsequent surgical biopsies, a change in the histological diagnosis was observed in a lesion with a higher biological risk in 26.5% of the cases (Table 1). With regard to HDAs, while the percutaneous biopsies diagnosed this type of lesion in 40.1% of patients; in the surgical biopsies, this percentage was reduced to 24% of the total. Only 32.2% of the percutaneous biopsies with a diagnosis of ADH maintained this diagnosis after surgical biopsy. In 6.8% of the ADHs, a progression was found in the histological diagnosis of a high-risk lesion, in 5.1% of ductal carcinomas *in situ* (DCISs) and in 1.7% of invasive ductal carcinomas (IDCs). On the other hand, in the case of FEAs, 25% presented a progression in histological diagnosis to a lesion of high biological risk, in 21.1% of ADHs and in 3.8% of CDISs, requiring a second treatment (hormone therapy prophylaxis in ADHs or surgery with free margins and radiotherapy in the case of CDISs).

## Discussion

Malignant mammary pathology has reached a new turning point in which, as a product of greater public screening, a remarkable increase has been experienced in the incidental discovery of suspicious lesions in breast imaging. The reduction in breast cancer mortality is owed as much to the implementation of the screening mentioned as it is to the improvement in treatments and our understanding of the disease.

BLAs as a finding in the percutaneous biopsies of radiologically suspicious lesions represent up to 10% of medical consultations in breast pathology care centres in the United States, in which they have a record of medical consultations by type of morbidity [4]. Its clinical confrontation and the decision to treat, operate or monitor are intensely debated subjects, on which a consensus still does not exist. Innumerable studies have been undertaken to try and elucidate this matter. Some have pointed to the genetic testing of ADH in order to predict its behaviour and eventual risk of developing into breast cancer [5]. For ADH, the literature reports a RR of 4–5 of developing breast cancer. There exists a recommendation, based on a review [6], that emphasises the importance of the radiopatholological correlation in BBD, so as to attain the best approach for its management. In the study, the authors conclude that, while existing studies have shown that surgery as a treatment for this type of lesion is not always necessary, there are too few multi-centred studies with a sufficient number of patients and follow-up, and that include benign breast lesions, to be able to define with greater clarity which lesions indeed confer a greater risk of developing breast cancer. Currently, the standard recommendation for the management of HDAs is surgical excision, particularly in cases associated with a mass-like lesion or discordance in radio-pathology is involved. We have tried to identify characteristics that might allow us to define subgroups of patients with favourable outcomes, in whom surgery could be omitted, considering the number of microcalcifications removed in the percutaneous biopsy, the absence of palpable lesions, the lesser involvement of the terminal duct lobular units and the absence of necrosis. The authors note that by means of evaluation on these criteria, the risk of missing a cancer diagnosis would be less than 5%. However, they recognise that the data are retrospective, for the most part, relying on only one prospective study among them [7]. Therefore, as long as these are not in the prior history, the recommendation for surgery continues to be the standard.

Among BLAs, FEA represents about 5% of percutaneous breast biopsies [8]. For FEA, the risk isn’t as clear as for ADH, with an RR of 1.5 or near 0 reported in breast cancer associated with its presence [7]. Other studies report that between 10% and 15%, and up to 30%, of patients with FEA presented with cancer after revision of the surgical specimen biopsy [2, 9, 10] which has led to suggesting surgical excision of these lesions after their diagnosis. In particular, one recently published study shows in its series that for the FEA, the risk of presenting invasive carcinoma in the surgical biopsy is 0%, while the risk of presenting with DCIS is 2.4%. While these risks are low, the risk of rising to a category of higher biological risk such as ADH, ALH or LCIS is close to 30%, which is why they argue that they may not be indicators of surgery or whether they would be good candidates for follow-up, always when they are not carriers of high-risk mutations [11].

Regarding the results of our study, it was observed that with the FEA, only 3.8% presented with cancer in the definitive surgical biopsy. A significant finding was the fact that up to 25% of patients diagnosed by percutaneous biopsy of BLA were observed to have a progression to a higher risk lesion. This is consistent with what was observed by Lamb, *et al* [11]. It is important to comment on the high discrepancy observed between the percutaneous biopsy and that of the BLA surgical specimen. An example of this is what was observed with ADH, where the total of percutaneous biopsies with findings of BLA, 40.1% corresponded to ADH, decreasing considerably when reviewing the surgical specimen biopsy, where only 24% of all BLA were found to correspond effectively to ADH. This discrepancy could be due to the fact that some ADHs are completely resected by the percutaneous needle or because in another atypical lesion identified in the percutaneous sample, ADH is subsequently detected in the surgical specimen. On the other hand, there is a lower progression in the diagnosis of ADH to a higher risk lesion (6.8%) that is usually described in the literature, where 15% to 30% of cases present a progression to DCIS or IDC [12, 13].

In mammary pathology, screening interventions and the treatment of benign findings, but with the risk of malignancy, seem to be a topic that ‘keeps coming up’. After several decades of promoting screenings, more studies and more treatment, we are getting to a point where the numbers show that, at least in the case of BLA, more treatment doesn’t necessarily mean a better prognosis. It is important to remember that the overdiagnosis of mammary lesions suspicious of malignancy implies a substantial social burden not only relative to economic and social costs but also in what it means for the patient and their family to get a diagnosis of a histopathological finding that can be associated with breast cancer, during a period of time when it is not easy or obvious to determine.

For the time being, to the extent that clinical studies support this, it is not possible to change course towards a plan to reduce over-treatment. However, it is possible to contribute at least in part and with the concurrence of various clinical series, to getting closer to generating a new consensus regarding the management of suspicious findings of malignancy.

## Conclusion

Given the increasing prevalence of BLA, it has become critical to clearly define the handling of these lesions. Our data show the clinical relevance of the ADH and FEA diagnoses in percutaneous biopsies. Currently, FEA is considered a lesion of low or almost no risk of developing a ductal carcinoma. However, in this study, it is evident that the diagnosis of said lesion obtained by percutaneous biopsy has the possibility of being associated with lesions of greater biological risk (ADH 21% and DCIS 4%) in a not insignificant percentage of cases (25%). Based on the data reported in this study, we recommend considering surgical excision in patients with breast lesions with a percutaneous biopsy compatible with FEA, especially when presented with other associated risk factors.

## Conflicts of interest

The authors of this study do not have any conflicts of interest.

## Figures and Tables

**Table 1.** Detail of histological findings in needle biopsies and their correlation with surgical biopsies (dark cells reflect the change of progression to a diagnosis of higher risk).

Needle biopsies (no. and %)		Surgical biopsy (no. and % depending on previous diagnosis)			Progression (%)
FEA	52 (35.4%)	FEA	31/52	59.6%	84.6%
		ADH	11/52	21.2%	
		DCIS	2/52	3.8%	
		FCM	6/52	11.5%	
		OA	1/52	1.9%	
		LCIS	1/52	1.9%	
ADH	59 (40.1%)	FEA	8/59	13.6%	
		ADH	19/59	32.2%	
		MFQ	6/59	10.2%	
		LCIS	22/59	37.2%	
		DCIS	3/59	5.1%	6.8%
		IDC	1/59	1.7%	
HLA	8 (5.4%)	ADH	1/8	12.5%	
		HLA	2/8	25.0%	
		MFQ	1/8	12.5%	
		LCIS	4/8	50.0%	
LCIS	13 (8.8%)	FEA	1/13	7.7%	
		LCIS	10/13	76.9%	
		MFQ	1/13	7.7%	
		ILC	1/13	7.7%	
OA	15 (10.2%)	FEA	1/15	6.7%	
		ADH	5/15	33.3%	
		DCIS	4/15	26.7%	
		MFQ	1/15	6.7%	
		OA	4/15	26.7%	
Total 147 (100%)					

FEA: flat epithelial atypia, ADH: atypical ductal hyperplasia, DCIS: ductal carcinoma in situ, FCM: fibrocystic mastopathy, OA: Other atypia, LCIS: lobular carcinoma in situ, IDC: invasive ductal carcinoma, ILC: invasive lobular carcinoma
